# Multi-Walled Carbon Nanotubes Augment Allergic Airway Eosinophilic Inflammation by Promoting Cysteinyl Leukotriene Production

**DOI:** 10.3389/fphar.2018.00585

**Published:** 2018-06-05

**Authors:** Sophia Carvalho, Maria Ferrini, Lou Herritt, Andrij Holian, Zeina Jaffar, Kevan Roberts

**Affiliations:** Center for Environmental Health Sciences, Department of Biomedical and Pharmaceutical Sciences, University of Montana, Missoula, MT, United States

**Keywords:** allergic airway inflammation, pulmonary eosinophils, multi-walled carbon nanotubes, cysteinyl leukotrienes, 5-lipoxygenase inhibitors, nanoparticles

## Abstract

Multi-walled carbon nanotubes (MWCNT) have been reported to promote lung inflammation and fibrosis. The commercial demand for nanoparticle-based materials has expanded rapidly and as demand for nanomaterials grows, so does the urgency of establishing an appreciation of the degree of health risk associated with their increased production and exposure. In this study, we examined whether MWCNT inhalation elicited pulmonary eosinophilic inflammation and influenced the development of allergic airway inflammatory responses. Our data revealed that instillation of FA21 MWCNT into the airways of mice resulted in a rapid increase, within 24 h, in the number of eosinophils present in the lungs. The inflammatory response elicited was also associated with an increase in the level of cysteinyl leukotrienes (cysLTs) present in the bronchoalveolar lavage fluid. CysLTs were implicated in the airway inflammatory response since pharmacological inhibition of their biosynthesis using the 5-lipoxygenase inhibitor Zileuton resulted in a marked reduction in the severity of inflammation observed. Moreover, FA21 MWCNT entering the airways of mice suffering from house dust mite (HDM)-elicited allergic lung inflammation markedly exacerbated the intensity of the airway inflammation. This response was characterized by a pulmonary eosinophilia, lymphocyte infiltration, and raised cysLT levels. The severity of pulmonary inflammation caused by either inhalation of MWCNT alone or in conjunction with HDM allergen correlated with the level of nickel present in the material, since preparations that contained higher levels of nickel (FA21, 5.54% Ni by weight) were extremely effective at eliciting or exacerbating inflammatory or allergic responses while preparations containing lower amounts of nickel (FA04, 2.54% Ni by weight) failed to initiate or exacerbate pulmonary inflammation. In summary, instillation of high nickel MWCNT into the lungs promoted eosinophilic inflammation and caused an intense exacerbation of pre-existing allergic airway inflammation by facilitating cysLT biosynthesis. These findings suggest that exposure to airborne MWCNT is likely to have adverse inflammatory effects in individuals suffering from atopic asthma and, in this context, further investigation of the therapeutic effects of pharmacological agents that block leukotriene synthesis is warranted.

## Introduction

The commercial demand for nanoparticle-based materials has expanded rapidly in recent years. As the industry grows, the likelihood of workers and consumers being exposed to airborne nanoparticles increases. Thus, assessment of any health risks associated with these materials will become increasingly important. Of the many types of engineered nanomaterials available, multi-walled carbon nanotubes (MWCNT) are among the most useful for a broad range of applications, including construction in the aerospace industry, electronics manufacture, and medical treatments. MWCNT possess excellent tensile strength and electrical conductivity, and can be easily functionalized for medical use (Bianco et al., 2005). However, the same properties that make MWCNT so useful also make them potentially hazardous. By design, MWCNT have high aspect ratios and, while only a few nanometers wide, may be several micrometers long (Ryman-Rasmussen et al., 2009). Inhalation represents a primary route of exposure of MWCNT because they are light and easily become airborne. As in the case of asbestos fibers, MWCNT are able to aggregate within the lung interstitium and are not easily degraded by alveolar macrophages. Health effects similar to asbestosis have been reported, including pulmonary fibrosis and granuloma formation, in addition to lung inflammatory responses and cytotoxicity (Girtsman et al., 2014; Wang et al., 2014). There have been significant advances in our understanding of MWCNT toxicity, yet the cellular and molecular events that underpin its capacity to evoke pulmonary inflammation remain unclear. Therefore, the aim of the present study is to determine the ability of MWCNT to elicit lung inflammation and exacerbate allergic airway inflammation, and to determine the role of cysteinyl leukotrienes (cysLTs) in this process.

Asthma is a complex and chronic inflammatory disorder of the airways that affects approximately 300 million people worldwide and continues to rise in both incidence and morbidity (Braman, 2006). As much as 85% of asthmatic patients are typically allergic to the common house dust mite (HDM) allergen (Gregory and Lloyd, 2011). Allergic asthma is characterized by airway eosinophilic inflammation, airway hyperreactivity (AHR), and mucus hypersecretion and remodeling, causing reversible airway obstruction. The pathological features of asthma are thought to be a consequence of the activation of Th2 cells and their associated cytokines including IL-4, IL-5, IL-9, and IL-13 (Finkelman et al., 2010). IL-13 is a key type 2 cytokine that orchestrates many of the features of airway inflammation and remodeling in human asthma. In addition, research in animal models of asthma have also provided evidence that cysLTs are important contributors to the development of eosinophilic inflammation and airway remodeling processes including mucus hypersecretion, collagen deposition, and lung fibrosis (Henderson et al., 2002; Holgate et al., 2003; Kanaoka and Boyce, 2004). In this study, we demonstrate that instillation of high nickel FA21 MWCNT into the lungs promoted the rapid onset of lung eosinophilic inflammation and intensely exacerbated existing allergic airway inflammation. The severity of these effects was shown to be highly dependent on the amount of nickel present in the material since low nickel MWCNT were poor at inducing lung inflammatory responses. The pulmonary eosinophilic inflammation elicited following FA21 MWCNT inhalation required the action of cysLTs, since this response was inhibited by treatment of mice with the 5-lipoxygenase inhibitor Zileuton.

## Materials and Methods

### Mice

C57BL/6 (The Jackson Laboratory, Bar Harbor, ME, United States) were used (10–16 weeks old) throughout this study. Mice were bred under pathogen-free conditions in a barrier facility and experimental animals maintained in micro-isolator cages and treated in accordance with National Institutes of Health guidelines and the American Association of Laboratory Animal Care regulations. All animal experiments were approved by the Institutional Animal Care and Use Committee (University of Montana), and performed according to the National Institute of Health guidelines.

### Intra-Tracheal Administration of MWCNT Preparations

The inflammatory response elicited by MWCNT entering the airways of naïve and HDM primed mice was examined using two different types of MWCNT, high nickel FA21 (5.54% nickel content, Sun Innovations, Inc., Fremont, CA, United States) and low nickel FA04 (2.54% nickel content, M K Impex Canada, Mississauga, ON, Canada). The particles were administered to adult C57BL/6 mice by oropharyngeal aspiration. The MWCNT were provided by Dr. Nigel Walker and Brad Collins at the National Toxicology Program (NTP), National Institute of Environmental Health Sciences, and previously characterized as described by Hamilton et al. (2012) and Girtsman et al. (2014). Purity and metal content of the MWCNT were determined using thermal gravimetric analysis (TGA) and X-ray fluorescence spectrometry, respectively. Diameter and agglomeration state of the MWCNT were determined by transmission electron microscopy (TEM) and dynamic light scattering (DLS), respectively. The physical characteristics of MWCNT FA21 and FA04 and their average agglomerate size measurements in dispersion media (DM) used in this study are shown in **Table 1**. FA21 formed fairly large agglomerate size of 469 nm in media which was between the two peaks (102 and 644 nm) observed for FA04 sample (Hamilton et al., 2012).

**Table 1:** Physical characteristics of MWCNT used in this study.

MWCNT	Nickel content	Diameter	Length	Agglomerate size	Agglomerate size
sample	(% total mass)	(nm)	(μm)	in water (nm)	in DM (nm)
FA21	5.54	27	5-15	429	469
FA04	2.54	33	5-15	682	102 and 644

Multi-walled carbon nanotubes (50 μg) free from endotoxin contamination, were suspended in DM that consisted of PBS containing 0.6 mg/ml mouse serum albumin (Sigma-Aldrich, St. Louis, MO, United States) and 0.01 mg/ml 1,2-dipalmitoyl-sn-glycero-3-phosphocholine (Sigma-Aldrich) generated using a cup-horn sonication for 1 min, as previously described (Girtsman et al., 2014). Mice were exposed to a MWCNT concentration (50 μg/25 g mouse or 2 mg/kg) previously used to assess the inflammatory response *in vivo* (Jessop and Holian, 2015). The dosage was established and selected based on the lowest amount of MWCNT able to reproducibly induce a robust lung inflammation *in vivo*. The MWCNT had minimal levels of endotoxin contamination, specifically 1 pg in 50 μg of FA21 and 55 pg in 50 μg of FA04 (Lonza BioResearch). To administer MWCNT, 30 μL of 1.67 mg/mL MWCNT suspension was instilled into C57BL/6 mice via oropharyngeal aspiration. Briefly, mice were lightly anesthetized with inhaled isoflurane, suspended from piano wire by the upper incisors and MWCNT suspension was then pipetted into the trachea. The level of pulmonary inflammation and fibrosis was examined after 24 h and 6 days.

### Sensitization of Mice to House Dust Mite (HDM) Prior to MWCNT Administration

**FIGURE 1:**
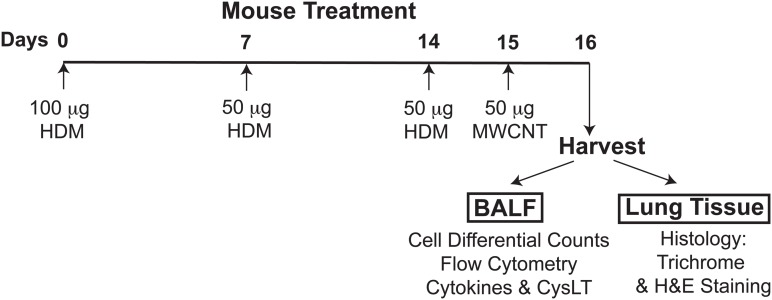
The experimental design of the study, timeline of allergen challenges and intratracheal MWCNT administration. To elicit allergic lung inflammation, C57BL/6 mice (4–6 per group) were given a sensitization dose of HDM intranasally (100 μg) on day 0, and then challenged with the allergen (50 μg) on days 7 and 14. To examine the proinflammatory properties of MWCNT, HDM-sensitized mice were additionally exposed to a single 50 μg dose of MWCNT by intratracheal instillation 24 h prior to harvest (on day 15). Control/sham-treated mice were given PBS and dispersion medium (in place of HDM or MWCNT, respectively). BALF and lung tissue were collected 24 h after MWCNT exposure (on day 16) for analysis of the lung inflammatory response.

To prime mice to HDM allergen and elicit allergic lung inflammation, animals were sensitized to HDM on day 0 by the intranasal administration of 100 μg of HDM allergen (*Dermatophagoides pteronyssinus*, Greer Laboratories) in 30 μl of PBS and then challenged on days 7 and 14 by intranasal treatment with 50 μg of HDM (30 μl total volume). HDM allergen preparations used throughout this study contained minimal levels of LPS. Control groups comprised mice receiving 30 μl of PBS on days 0, 7, and 14. To determine the level of mucosal inflammation, bronchoalveolar lavage fluid (BALF) and lung tissue were harvested 48 h after the last challenge on day 16. To examine the proinflammatory properties of MWCNT and their effect on allergic airway inflammation, mice were additionally challenged with a single 50 μg dose of MWCNT (30 μl of 1.67 mg/ml suspension) administered oropharyngeally 24 h prior to harvest, as illustrated in **Figure 1**.

### Evaluating the Level of Pulmonary Inflammation

Mice were euthanized 24 h or 6 days post-MWCNT instillation and bronchoalveolar lavage was performed to collect BALF for analysis. Eosinophil peroxidase (EPO) levels in the lavage cells were determined by colorimetric analysis using orthophenylene diamine dihydrochloride as detailed previously (Lee et al., 1999). Cell differential percentages were determined by light microscopic evaluation of Hema 3-stained cytospin preparations and expressed as absolute cell numbers. Lung tissue was collected for histological examination using H&E (peribronchial inflammation) and Trichrome (lung fibrosis) staining.

### Measurement of Cytokines and CysLT Production

Bronchoalveolar lavage fluid cytokine levels were determined using Quantikine ELISA (R&D Systems, Minneapolis, MN, United States) and CX3CL1 chemokine levels measured using DuoSet ELISA (R&D Systems). CysLT levels in the BALF were measured using ELISA (Cayman Chemical, Ann Arbor, MI, United States) according to manufacturer’s instructions.

### Histological Determination of Lung Inflammation and Collagen Deposition

Lung tissue was fixed in 4% paraformaldehyde and embedded in paraffin using a Leica ASP 300 tissue processor (Leica, Bannockburn, IL, United States). Microtome sections were cut at 5 μm thickness and stained with hematoxylin and eosin (H&E) using a Shandon Varistain 24-4 (Thermo Fisher Scientific). In addition, sections were stained using Gomori’s Trichrome (EMD Chemicals, Gibbstown, NJ, United States) for histological analysis using a Thermo Shandon automated stainer. The level of pulmonary or peribronchial inflammation (H&E stain) and collagen deposition (Trichrome stain) was analyzed by microscopy and the transmitted light images were collected on a Nikon Eclipse 800 microscope equipped with an Olympus DP 26 camera and cellSens software (Version 1.9).

### Flow Cytometric Analysis of Inflammatory Cells in the BALF

Bronchoalveolar lavage fluid cells were collected (from 4–6 mice per group) and the pooled cells were FcγR blocked using 2.4G2 antibody (ATCC) and stained with combinations of the following mouse conjugated mAb: allophycocyanin (APC) or FITC anti-CD3, APC/Cy7 anti-CD4, PE anti-CD8a, APC/Cy7 anti-Ly-6G/Ly6C (Gr-1), PE, FITC or Brilliant Violet 421 anti-CD11b, APC or PE anti-F4/80 (all purchased from BioLegend, San Diego, CA, United States). In addition, PE anti-Siglec-F (BD Biosciences) was used to stain eosinophils. Flow cytometric acquisition was performed on a FACSAria II (BD Biosciences) by 4-color analysis using FACSDiVa software and FlowJo, with a minimum of 50,000 live, single-cell events per sample collected. Cells were live gated based on Forward versus Side Scatter properties. In addition, isotype controls were used to control for non-specific background antibody binding. To this end, cells were stained using conjugated (APC, FITC, PE, APC/Cy7, or Brilliant Violet 421) control rat IgG2a, rat IgG2b, and hamster IgG1 antibody (obtained from Becton Dickinson).

### Treatment of Mice With Zileuton to Inhibit MWCNT-Induced Inflammation

C57BL/6 mice were treated with either vehicle (0.5% methyl-cellulose/0.2% Tween 80 in water) or Zileuton, a 5-lipoxygenase inhibitor (10 mg/Kg, Tocris Bioscience, Minneapolis, MN, United States), by oropharyngeal instillation 3 h before administration of FA21 MWCNT (50 μg). Zileuton treatment was based on the concentration (10 mg/Kg) and protocol used previously by Clarke et al. (2014). Control mice comprised of mice treated with carrier alone. BALF was collected 24 h after MWCNT exposure and the level of inflammation elicited was evaluated by monitoring EPO and cysLT levels.

### Statistical Analysis

Data were analyzed using GraphPad Prism 5.0 (GraphPad, La Jolla, CA, United States). Results involving two variables were analyzed by two-way ANOVA with a Bonferroni *post hoc* test. Data comparing two groups were analyzed using an unpaired *t*-test. Figures show combined data from multiple studies or independent repeats (two or more). Data shown are mean ± SEM. A *p*-value < 0.05 was considered statistically significant. Significance denoted by ^∗^, ^∗∗^, or ^∗∗∗^ is defined as *p* < 0.05, *p* < 0.01, or *p* < 0.005, respectively.

## Results

### Characterization of the Inflammatory Response Elicited Following MWCNT Instillation

**FIGURE 2:**
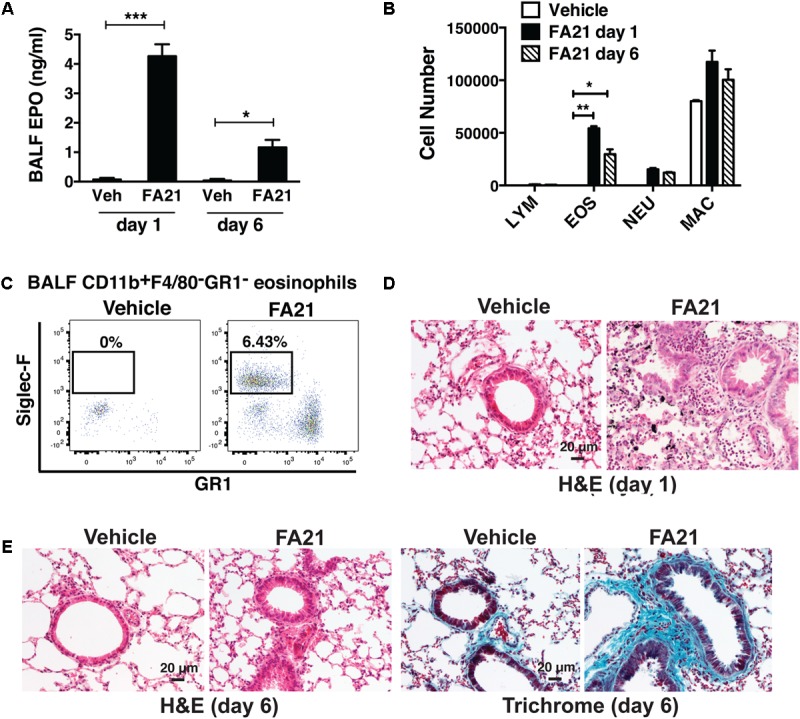
Intratracheal administration of high nickel MWCNT elicited pulmonary eosinophilic inflammation. To examine the inflammatory properties of high nickel MWCNT, 50 μg of FA21 was administered to C57BL/6 mice (4–6 mice per group) by oropharyngeal instillation (30 μl). Controls comprised of mice treated with dispersion medium alone (vehicle). To examine the resultant inflammatory response, Bronchoalveolar lavage fluid (BALF) and lung tissue were collected for analysis 1 and 6 days after FA21 administration. **(A)** Eosinophil peroxidase (EPO) activity expressed by BALF cells collected 1 or 6 days after FA21 or vehicle exposure was determined by colorimetric assay. **(B)** BALF cell differential counts were determined and expressed as absolute cell numbers per mouse of lymphocytes (LYM), macrophages (MAC), eosinophils (EOS), and polymorphonuclear neutrophils (NEU). Results are mean ± SEM (*n* = 6), ^∗^*p* < 0.05, ^∗∗^*p* < 0.01, and ^∗∗∗^*p* < 0.001. **(C)** CD11b^+^F4/80^-^GR1^-^Siglec-F^+^ eosinophil numbers present in the BALF collected 24 h after vehicle or FA21 exposure was determined by flow cytometry. **(D)** Pulmonary inflammation was determined by histological analysis of H&E-stained lung tissue collected 24 h after vehicle or FA21 exposure (20×). Lung tissue images from FA21-exposed mice show evidence of MWCNT agglomerates (appear as black aggregates). **(E)** Pulmonary inflammation and collagen deposition in lung tissue collected 6 days after vehicle or FA21 exposure was assessed by histological analysis of H&E- and Trichrome-stained segments, respectively (20×). Data are representative of 4 independent experiments.

The two different types of MWCNT compared in this study were high nickel FA21 and low nickel FA04. Both particles have been previously characterized (Hamilton et al., 2012) and shown to possess several comparable physical characteristics including similar specified length (5–15 μm) and diameter (27–33 nm). To discern their potential inflammatory properties, MWCNT or vehicle alone (control) was instilled directly into the airways of C57BL/6 mice by oropharyngeal administration and evidence of an inflammatory response was examined. A single instillation of 50 μg of FA21 into the airways elicited a rapid lung inflammatory response within 24 h (**Figure 2**). The BALF recovered from mice receiving FA21 instillations contained significantly elevated numbers of eosinophils that were detected by increased levels of cell-associated EPO (**Figure 2A**) and confirmed by cell differential counts (**Figure 2B**). Both the level of EPO and the number of eosinophils in the airways remained raised for 6 days after a single instillation of the MWCNT. While no evidence of lymphocyte infiltration was observed, increased numbers of neutrophils and macrophages were also observed in the BALF following FA21 MWCNT exposure, although this did not reach statistical significance. To quantify cellular populations present in the airways after the MWCNT exposure, inflammatory cells present in the BALF were also analyzed by flow cytometry (**Figure 2C**), as previously described (Simons et al., 2017). Eosinophils were identified using a gating strategy in which leukocytes were first selected by FSC and SSC, and CD11b^++^/F4/80^-^ cells were further characterized by the degree of Siglec-F and GR1 expression. Eosinophils were identified by high expression of Siglec-F (by GR1 negative cells). Alveolar macrophages were excluded on the basis of F4/80 staining. This process revealed that FA21 caused a marked increase in the number of Siglec-F^+^ eosinophils in the BALF (mean of 54,340 ± 1965 cells, *n* = 4), which was evident 24 h after exposure, and that these cells were absent in the airways of unexposed (vehicle control) mice (**Figure 2C**). Remarkably, histological analysis of lung tissue using H&E staining showed evidence of pronounced lung/peribronchial inflammation 24 h after FA21 MWCNT inhalation (**Figure 2D**) and this response was markedly diminished 6 days after exposure (**Figure 2E**). Vehicle-treated control mice did not develop any pulmonary inflammation (**Figures 2D,E**). Moreover, trichrome staining exhibited increased deposition of collagen in the lungs of FA21-treated mice compared to control animals providing evidence of fibrogenesis just 6 days after MWCNT exposure (**Figure 2E**). Notably, the lungs of mice treated with FA21 displayed evidence of MWCNT agglomerates which appear as black aggregates close to the site of inflammation (**Figure 2D**).

### A Role for CysLTs in Promoting MWCNT-Induced Inflammation

**FIGURE 3:**
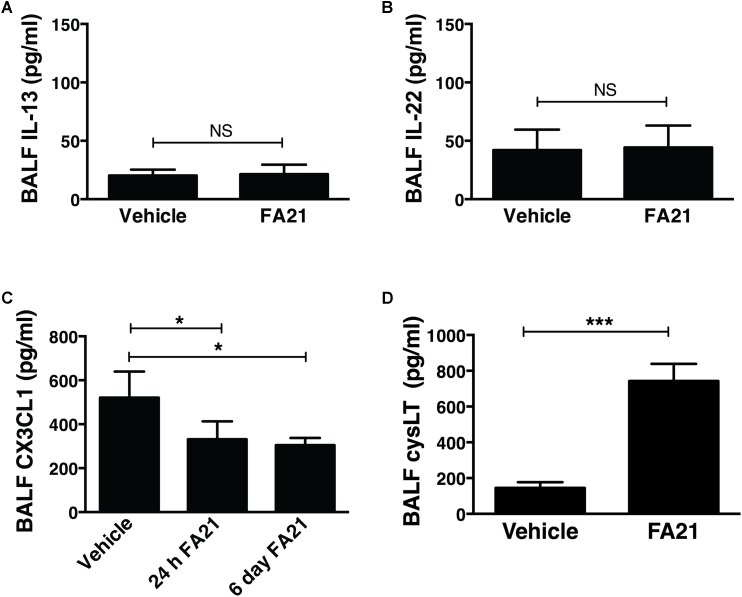
Intratracheal administration of high nickel MWCNT resulted in the biosynthesis of cysLT in the absence of T cell-associated cytokines. C57BL/6 mice (six per group) were exposed to FA21 MWCNT (50 μg) or dispersion medium (vehicle alone) by oropharyngeal instillation. BALF was collected 24 h after exposure and the levels of IL-13 **(A)**, IL-22 **(B)**, CX_3_CL1 **(C)**, and cysLT **(D)** determined by ELISA. Results are mean ± SEM (*n* = 6), ^∗^*p* < 0.05 and ^∗∗∗^*p* < 0.001 (NS = not significant). Data are representative of 3–4 independent experiments.

**FIGURE 4:**
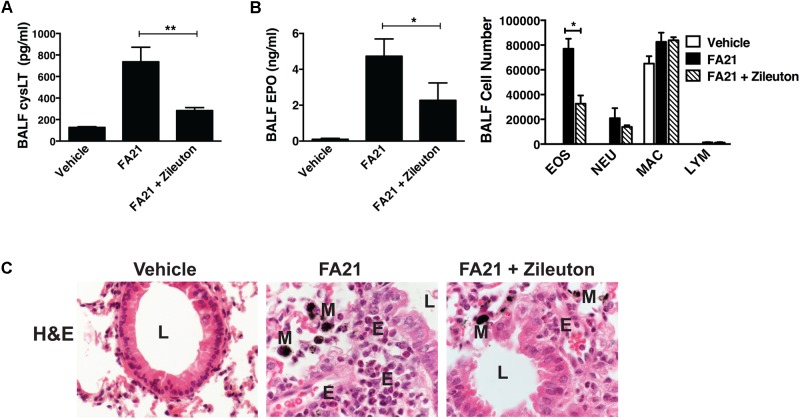
Pharmacological inhibition of cysLT biosynthesis using a 5-lipoxygenase inhibitor caused a reduction in MWCNT-induced pulmonary eosinophilic inflammation. To examine whether cysLTs contributed to FA21 MWCNT-induced inflammation, C57BL/6 mice (six per group) were treated with either Zileuton (10 mg/Kg) or vehicle by oropharyngeal instillation 3 h before administration of FA21 MWCNT (50 μg intratracheally). Controls comprised of mice treated with carrier alone. BALF and lung tissue was collected 24 h after MWCNT exposure. **(A)** The level of cysLT in the BALF was evaluated using ELISA. **(B)** BALF cell-associated eosinophil peroxidase (EPO) activity was assessed by colorimetric assay. Cell differential counts were determined and expressed as absolute cell numbers per mouse of eosinophils (EOS), polymorphonuclear neutrophils (NEU), macrophages (MAC) and lymphocytes (LYM). Results are mean ± SEM (*n* = 6), ^∗^*p* < 0.05 and ^∗∗^*p* < 0.01. **(C)** Peribronchial inflammation was assessed by histological analysis of lung tissue using H&E staining (60×) (E, eosinophils; M, MWCNT aggregates; L, bronchial lumen). Data are representative of three independent experiments.

Recent evidence indicates that Th2 cytokines (such as IL-5 and IL-13) and other T-cell associated cytokines (such as IL-22 and IL-17A) are involved in allergic airway inflammation in asthma (Hirose et al., 2017). In this study, although FA21 promoted the rapid onset of an eosinophil recruitment to the airways, surprisingly both the proinflammatory Th2 cytokines IL-13 and IL-5, as well as IL-22 and IL-17 were universally absent from the BALF of FA21-exposed mice, as evidenced by lack of increase in the levels of IL-13 (**Figure 3A**), IL-22 (**Figure 3B**), IL-5, and IL-17A (data not shown). The chemokine CX_3_CL1 plays a key role in tissue recruitment of monocytes and NK cells and serves an important role in homeostatic and inflammatory states (Robinson et al., 2003; Zhang and Patel, 2010). While CX_3_CL1 was constitutively present in the BALF of control C57BL/6 mice, FA21 instillation caused a significant reduction in the level of this chemokine within 24 h and 6 days after exposure (**Figure 3C**). Given that eosinophils were the principal inflammatory cell present in airways of FA21-exposed mice and there is lack of Th2 or other cytokine production, the synthesis of other eosinophil chemotactic and survival factors was investigated. It has been previously reported that cysLTs can promote eosinophil migration (Luna-Gomes et al., 2013) and play an essential role during allergic lung inflammation (Henderson et al., 1996). Our experiments showed that only low levels of cysLTs were present in the BALF of control mice that were instilled with vehicle. In sharp contrast, the administration of FA21 resulted in a marked increase within 24 h in the level of cysLTs in the BALF (from 145.5 pg/ml in vehicle challenged to 742.8 pg/ml in response to MWCNT exposure) (**Figure 3D**). Since eosinophils can themselves produce cysLTs (Hirata et al., 1990), it was important to resolve whether the raised cysLT levels were simply a result of elevated numbers of eosinophils or whether the mediator caused their accumulation into the airway. To examine the latter possibility, the effect of blocking leukotriene biosynthesis was examined by treating mice with Zileuton, an inhibitor of 5-lipoxygenase (5-LO). Zileuton has been shown to block the biosynthesis of cysLT (LTC_4_, LTD_4_, and LTE_4_) and LTB_4_, which are products of the 5-LO pathway (Smith et al., 1995; Gaber et al., 2007). By shunting available arachidonic acid toward the cyclooxygenases, COX-1 and COX-2, the drug may result in raised prostaglandin production (Smith et al., 1995). It is also a weak inhibitor of cytochrome P450 activity but generally has few “off-target” effects (Lu et al., 2003). To investigate the effect of this drug, mice exposed to FA21 MWCNT were pre-treated with Zileuton (10 mg/Kg) 3 h prior to oropharyngeal instillation of FA21 (50 μg) or vehicle and the level of pulmonary inflammation and cysLTs present in BALF was determined 24 h after exposure. Interestingly, pretreatment of FA21-exposed mice with Zileuton caused a significant reduction in cysLT levels present in the BALF by approximately 60% (**Figure 4A**, *p* < 0.01). This inhibition in cysLT synthesis was coincident with a corresponding reduction in the number of eosinophils and cell-associated EPO levels in the BALF (**Figure 4B**) as well as diminished peribronchial eosinophilic inflammation evident on histological examination (**Figure 4C**), suggesting that MWCNT-induced leukotrienes (cysLTs and possibly LTB_4_) promoted eosinophil accumulation in the airways.

### Exacerbation of HDM-Induced Allergic Lung Inflammation by MWCNT

**FIGURE 5:**
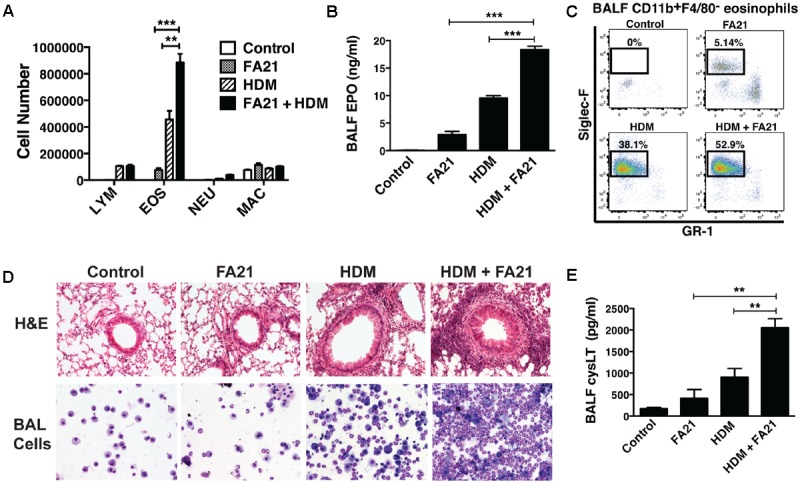
Exposure to FA21 MWCNT exacerbates HDM-induced allergic lung inflammation and promotes cysLT production. To examine the effect of MWCNT exposure on pre-existing allergic airway inflammation, C57BL/6 mice (4–6 per group) were intranasally challenged with PBS (control) or HDM allergen over a 2-week period (on days 0, 7, and 14) to induce allergic inflammation and then exposed to FA21 MWCNT (50 μg) on day 15 by intratracheal administration. BALF and lung tissue were collected 24 h after MWCNT administration on day 16 for analysis of pulmonary inflammation (as illustrated in the experimental design in **Figure 1**). Groups comprised of mice treated with FA21, HDM, or FA21 + HDM. Control mice were treated with carrier alone. **(A)** BALF cell differential counts were determined and expressed as absolute cell number per mouse of lymphocytes (LYM), eosinophils (EOS), macrophages (MAC), and polymorphonuclear neutrophils (NEU). **(B)** Cell-associated EPO levels in the BALF were determined by colorimetric assay. **(C)** CD11b^+^F4/80^-^GR1^-^Siglec-F^+^ eosinophil numbers present in the BALF were analyzed by flow cytometry. **(D)** Peribronchial and perivascular inflammation was assessed by histological analysis of lung tissue sections stained with H&E (20×). In addition, inflammatory cells infiltrating the airways were examined by microscopic evaluation and imaging of cytospin preparations of BALF cells stained using Hema3. **(E)** CysLT levels present in BALF determined by ELISA. Results are mean ± SEM (*n* = 6), ^∗∗^*p* < 0.01 and ^∗∗∗^*p* < 0.001. Data are representative of three independent experiments.

At the time points examined, the level of eosinophilic inflammation elicited by FA21 MWCNT alone was markedly less severe than that typically elicited by known allergens like HDM. This is likely a consequence of the low numbers of circulating (blood borne) eosinophils present in a non-allergic animal. To assess the potential of MWCNT to exacerbate airway inflammation in allergen-sensitized animals, a single 50 μg dose of FA21 was administered to mice on the final day of our murine model of allergic asthma. The experimental design, timeline of HDM challenges and MWCNT exposures of C57BL/6 mice are illustrated in **Figure 1**. Given that HDM is a globally ubiquitous allergen to which as many as 85% of asthmatics react (Gregory and Lloyd, 2011), our model uses a series of three doses of HDM over 2 weeks to sensitize mice and prompt an allergic response. Observed inflammatory responses correlate with the findings of our previous studies (Simons et al., 2017), with mice sensitized to HDM displaying significantly elevated eosinophilia and cysLT production compared to sham-treated mice (**Figures 5A–E**). Interestingly, mice that were sensitized to allergen and further exposed to FA21 MWCNT showed a pronounced increase in pulmonary inflammation compared to mice given only HDM allergen or only FA21. BALF differential cell counts revealed a clear influx of eosinophils into the airways, most notably in mice exposed to both HDM allergen and FA21 (**Figure 5A**). Alveolar macrophages and lymphocytes were also present but in much lower numbers (**Figure 5A**). Specifically, FA21 exposure of mice challenged with HDM allergen did not result in any significant changes in the number of BALF CD4^+^ or CD8^+^ T cells when compared to mice exposed to HDM only (Supplementary Figure 1). As before, cell-associated EPO measurements confirmed the presence of a considerable eosinophil population in the airways of HDM challenged mice that inhaled FA21 MWCNT (**Figure 5B**). BALF was further analyzed by flow cytometry; this revealed a striking increase in the airway Siglec-F^+^ eosinophil population of mice treated with HDM and FA21 (dual exposure) over those exposed to HDM alone or FA21 alone (**Figure 5C**). This exacerbated eosinophilic inflammatory response can be visualized quite clearly in histological images of lung tissue (H&E) or images of BAL cells harvested from allergen-challenged mice just 24 h after MWCNT instillation (**Figure 5D**). The dual-exposure (HDM + FA21) also elicited significantly greater degree of cysLT biosynthesis than did either HDM or FA21 exposure alone (**Figure 5E**). Together, these results demonstrate that the MWCNT inhalation strongly exacerbates the progression of allergic inflammatory responses in the lung.

### Comparison Between the Degree of Inflammation Elicited and the Nickel Content of MWCNT

**FIGURE 6:**
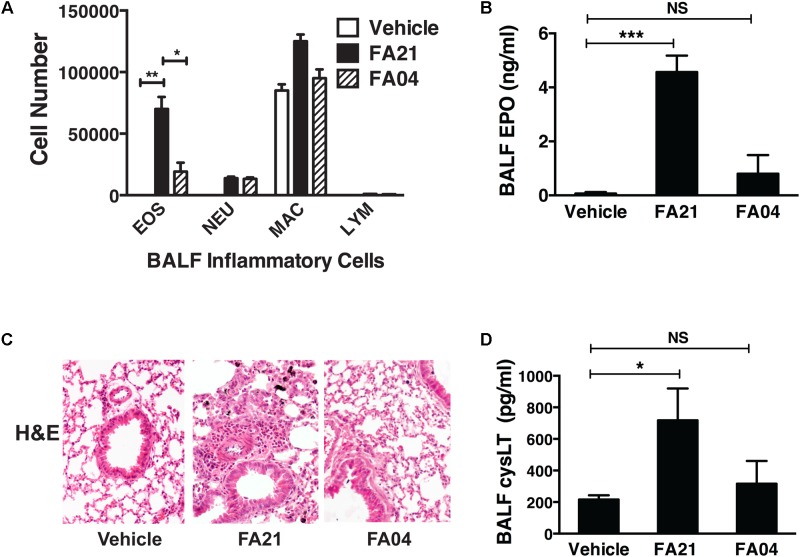
Comparison of the level of pulmonary inflammation elicited by exposure to MWCNT with high or low nickel content. The effect of nickel on the inflammatory process was investigated by comparing the airway inflammation elicited by exposure to MWCNT with high (FA21) or low (FA04) nickel content. C57BL/6 mice (four mice per group) were given vehicle alone (control), FA21 or FA04 (50 μg of MWCNT) by oropharyngeal instillation into the lungs and BALF was collected 24 h after exposure. **(A)** The level of pulmonary inflammation was determined by BALF cell differential counts and expressed as absolute cell numbers per mouse of lymphocytes (LYM), macrophages (MAC), eosinophils (EOS), and polymorphonuclear neutrophils (NEU). **(B)** BALF cell-associated EPO activity was measured using colorimetric analysis. **(C)** Lung inflammation was determined by histological analysis of H&E-stained tissue collected 24 h after vehicle, FA21 or FA04 exposure (10×). **(D)** CysLT levels in the BALF determined by ELISA. Results are mean ± SEM (*n* = 4), ^∗^*p* < 0.05, ^∗∗^*p* < 0.01, and ^∗∗∗^*p* < 0.001. Data are representative of three independent experiments.

**FIGURE 7:**
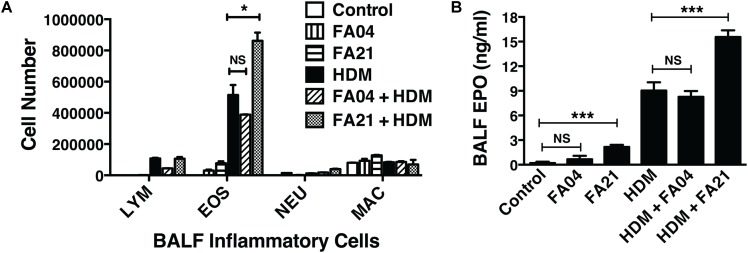
The effect of exposure to MWCNT with high or low nickel content in exacerbating allergic lung inflammation. To compare the effects of high (FA21) or low (FA04) nickel MWCNT exposure on allergic airway inflammation, C57BL/6 mice (four per group) were intranasally challenged with PBS (control) or HDM allergen over a 2-week period (on days 0, 7, and 14) to induce allergic inflammation and then exposed to vehicle, FA21 or FA04 (50 μg of MWCNT) on day 15 by intratracheal administration (illustrated in **Figure 1**). BALF was collected 24 h after MWCNT administration on day 16 for analysis of the airway inflammatory response. Groups comprised of mice treated with FA21 alone, FA04 alone, HDM alone, FA21 + HDM, FA04 + HDM, and carrier alone (control). **(A)** Cell differential counts in the BALF were determined and expressed as absolute cell numbers (per mouse) of lymphocytes (LYM), eosinophils (EOS), polymorphonuclear neutrophils (NEU), and macrophages (MAC). **(B)** The level of eosinophil infiltration was determined by measuring BALF cell-associated EPO using colorimetric analysis. Results are mean ± SEM (*n* = 4), ^∗^*p* < 0.05 and ^∗∗∗^*p* < 0.001. Data are representative of three independent experiments.

To compare the pro-inflammatory properties of high nickel FA21 with low nickel F04 MWCNT, each of these materials was administered to the lungs of C57BL/6 mice and the inflammation elicited was assessed. Previous analysis of the physical properties of FA21 and FA04 materials revealed that both form agglomerates (**Table 1**) with the major contaminant in both being nickel (comprising of 5.54 and 2.54%, respectively) (Hamilton et al., 2012). As detailed previously (Hamilton et al., 2012), oropharyngeal instillation of 50 μg of FA21 MWCNT into the lungs of naïve C57BL/6 mice elicited a strong inflammatory response (evident after 24 h of exposure) when compared to control (vehicle-treated) mice. Specifically, FA21 caused a marked elevation in the number of eosinophils in the airways which were detected by cell differential counts (**Figure 6A**) and confirmed by increased levels of cell-associated EPO in the BALF (**Figure 6B**). In marked contrast to FA21, the instillation of an equivalent amount of FA04 caused only a weak inflammatory response (**Figure 6A**) and the particle failed to elicit a significant increase in cell-associated EPO activity (**Figure 6B**). Both MWCNT FA21 and FA04 caused a slight increase in the number of neutrophils and macrophages in the BALF (evident 24 h after exposure) that did not reach statistical significance (**Figure 6A**). In addition, histological analysis of lung tissue using H&E staining showed evidence of pronounced lung tissue inflammation 24 h following FA21 exposure but not after FA04 inhalation (**Figure 6C**). Importantly, mice exposed to FA21 displayed noticeably higher cysLT levels in the BALF compared to mice given FA04 (**Figure 6D**). Consistent with the eosinophilic response, FA04 instillation failed to raise BALF cysLT levels significantly above what was found in the vehicle treated control group (**Figure 6D**). To further investigate the biological properties of these two materials, their pro-inflammatory effects were compared in mice that had been sensitized and challenged with HDM allergen. HDM allergen alone elicited an airway eosinophilia that was at least fivefold higher than that caused by FA21 MWCNT administration alone (**Figure 7A**). In HDM challenged animals, high nickel FA21 instillation 24 h prior to harvesting BALF resulted in a marked exacerbation of the level of pulmonary eosinophils present as determined by cell differential counts and cell-associated EPO activity (**Figures 7A,B**). In marked contrast, low nickel FA04 exposure of HDM challenged mice did not cause a significant increase in airway eosinophilic inflammation (i.e., similar levels of pulmonary eosinophilia was detected in HDM + FA04 group and HDM alone group, **Figure 7**).

## Discussion

As a consequence of the growth of the nanotechnology industry, there has been a striking increase in the types and quantity of MWCNT currently in production. This trend raises the likelihood of human exposure to these particles and necessitates a better appreciation for their potential impact on human health. The health risks arising from the respiration of MWCNT particles are of concern since this material is fibrous and bears several characteristics displayed by asbestos. MWCNT entering the airways of mice have been shown to elicit several pro-inflammatory events that originate from their activation of the NLRP3 inflammasome (Hamilton et al., 2012) and are associated with the induction of IL-6, TNF-α, and IL-1β (Hamilton et al., 2013), formation of granulomas and development of airway fibrosis (Inoue et al., 2009; Ryman-Rasmussen et al., 2009; Wang et al., 2011). MWCNT exposure also causes epithelial damage (Hamilton et al., 2012, 2013; Ohba et al., 2014) by a process involving COX-2 (Sayers et al., 2013) and is associated with epigenetic regulatory events arising from changes in global methylation patterns (Brown et al., 2016). Our study evaluated the inflammatory and fibrotic responses elicited by inhaled MWCNT particles and investigated whether such exposures exacerbated pre-existing allergic lung inflammation. Strikingly, the entry of FA21 MWCNT into the lungs of naïve mice caused a rapid recruitment (within 24 h) of CD11b^+^F4/80^-^GR1^-^ eosinophils into the airways. The increase of eosinophil numbers in the airways remained raised for 6 days after a single instillation of the MWCNT. Moreover, histological analysis of lung tissue revealed a marked pulmonary and peribronchial inflammation (after 24 h of FA21 exposure) as well as increased deposition of collagen consistent with the onset of fibrosis in the lungs after just 6 days of FA21 administration. Interestingly, the inflammatory response elicited by FA21 MWCNT revealed no detectable production of Th2 cytokines (IL-13 and IL-5) or other T-cell associated cytokines. However, a marked increase in the level of cysLTs was observed in the BALF within 24 h after a single instillation of FA21. Leukotrienes are mediators derived from arachidonic acid by the action of 5-LO. The cysLTs are a family of lipid mediators (LTC_4_, LTD_4_, and LTE_4_) produced by eosinophils, mast cells, basophils, dendritic cells, and macrophages that act through two structurally divergent G protein receptors, termed CysLT_1_ and CysLT_2_. The parent cysLT, LTC_4_, is synthesized by and released from these cells and converted to the airway constrictor LTD_4_ and ultimately to the stable metabolite, LTE_4_ (Kanaoka and Boyce, 2004; Laidlaw and Boyce, 2012; Luna-Gomes et al., 2013). CysLTs play an important role in the exacerbation of allergen-induced inflammation and airway remodeling and are known to facilitate lung eosinophil recruitment in both man and mouse (Holgate et al., 2003; Kim et al., 2006; Luna-Gomes et al., 2013). CysLT_1_ receptor mediates bronchoconstriction and eosinophil recruitment and adhesion molecule expression, while activation of CysLT_2_ induces vascular permeability and fibrosis (Fregonese et al., 2002; Saito et al., 2004; Peters-Golden and Henderson, 2007). In the current study, the induction of cysLT biosynthesis following inhalation of MWCNT played a central role in the inflammatory process since inhibition of leukotriene production, using Zileuton, markedly suppressed the level of pulmonary eosinophilia. Leukotriene modifiers, such as Montelukast (which specifically targets CysLT_1_) and Zileuton (a competitive inhibitor of 5-LO), are important anti-inflammatory drugs that are used for the treatment of atopic asthma (Dube et al., 1999) or as combination therapy in asthma. Our study suggests that a proven safe and effective drug can reverse some of the damaging effects arising from exposure to MWCNT. Since blockade of 5-LO action results in inhibition of eosinophils, this suggests that the 5-LO pathway plays a critical role in the development of airway inflammation.

While the inflammatory response elicited by FA21 MWCNT alone was present for several days, comparison of this response revealed that it was markedly weaker than that elicited following inhalation of HDM allergen. Triggers for eosinophilic inflammation can either be non-allergic or allergic in nature, with the former thought to be associated with activation of type 2 innate lymphoid cells (ILC2s) and the latter involving Th2 cells (Brusselle et al., 2013). A second consideration is whether eosinophils present in the airways derive from cells resident in the lung tissue or are recruited from the blood (e.g., by IL-5). With respect to the former scenario, resident “steady-state” eosinophils in normal non-inflamed intestinal and lung mucosa have been reported previously (Nussbaum et al., 2013), and a population of lung-resident Siglec^int^CD62L^+^ eosinophils have been described that are IL-5-independent with a ring-shaped nucleus (Mesnil et al., 2016). Our study raises the possibility that MWCNT inhalation causes the recruitment of steady-state eosinophils into the airspaces by facilitating the rapid production of cysLT, and that cysLTs play a crucial in promoting the migration of steady-state eosinophils into the airways.

Although FA21 MWCNT exposure was effective at promoting pulmonary eosinophilic inflammation in naïve mice, it was important to compare the magnitude of this response to that elicited by a common clinically relevant airborne allergen such as HDM. HDM allergen elicited a pulmonary inflammation following inhalation without the need to resort to the use of immunological adjuvants (Jaffar et al., 2007). Repeated inhalation of HDM allergen results in the activation of both innate and adaptive immunity characterized by recruitment of CD4^+^ Th2 cells, generation of specific IgE and infiltration of eosinophils into the airway (Wills-Karp et al., 2010), processes that typify the disease process in atopic asthma. In addition, it was important to resolve whether MWCNT exacerbated “pre-existing” allergic lung inflammation. This was particularly relevant given the evidence that FA21 MWCNT promotes eosinophil recruitment and would be expected to increase the number of eosinophils entering the airways of mice suffering from allergic lung inflammation. Our data revealed that instillation of FA21 into the airways of HDM challenged mice dramatically increased the number of eosinophils entering the airways by approximately twofold and markedly exacerbated the intensity of peribronchial and perivascular inflammation when compared to mice treated with HDM only. Type 2 cytokine production by both CD4^+^ T cells and ILC2s are thought to contribute to the inflammatory responses elicited by inhaled allergens (Scanlon and McKenzie, 2012; Gold et al., 2014; Christianson et al., 2015; Everaere et al., 2016). However, MWCNT administration did not appear to influence IL-13 and IL-5 production, or T cell responses since we did not observe any significant changes in the number of CD4^+^ or CD8^+^ T cells in the airways of allergen-challenged mice exposed to FA21. As previously detailed, the exposure of mice to HDM resulted in raised levels of cysLTs present in the BALF and these levels were markedly enhanced (by approximately twofold) 24 h after instillation of FA21 into the lungs. The exacerbation of the allergic inflammatory response by high nickel MWCNT suggests that exposure to these materials may have profound health consequences in individuals suffering from atopic asthma. This effect likely stems from the innate ability of such MWCNT to elicit rapid cysLT release and the contribution of this mediator to inducing airway eosinophilic inflammation. The effectiveness of these materials to markedly exacerbate existing allergic inflammation may prove more problematic than their ability to initiate a mild eosinophilic inflammation in normal individuals. Since high nickel MWCNT administration caused an intense exacerbation (twofold increase) of allergic airway inflammation within 24 h without enhancing Th2 responses, it is likely that FA21, by promoting cysLT, facilitates the migration of eosinophils from the lung tissue into the airspaces, rather than directly increasing circulating eosinophils entering the lungs. These results are in agreement with previous reports demonstrating that repeated administration of MWCNT augments allergic lung inflammation (Inoue et al., 2009, 2010), an effect that is intensified in mice lacking cyclooxygenase-2 (Sayers et al., 2013). Notably, in these mice, the absence of cyclooxygenase-2 would be expected to result in increased cysLT production.

Our data revealed that dramatically lower eosinophilic responses were elicited following the instillation of FA04 MWCNT compared to mice given FA21. The former preparation is known to contain significantly less nickel, implying that the difference in the inflammatory capacity of these two MWCNT may be a consequence of their nickel content. In the course of these studies, the low nickel FA04 MWCNT neither elicited a pulmonary eosinophilia when administered alone, nor exacerbated the allergic inflammatory response in mice that had been previously sensitized to HDM allergen. The observation that nickel is required for eliciting an eosinophilic response in the airway is intriguing and suggests that either the nickel itself, or changes in the physiochemical properties of the particles created by the raised levels of nickel had profound effects on several physical properties of the particles such as their respective agglomerate sizes (**Table 1**). It is possible that the difference in inflammatory capacity of the two MWCNT preparations could, in part, be due to differences in agglomerate size. In this context, the dispersion status of nickel-oxide nanoparticles was reported to affect their inflammatory response, with smaller agglomerates being more bioactive (Sager et al., 2016). However, in our study, FA04, which forms a smaller agglomerate size peak than FA21 (and might be expected to be more bioactive), elicited a weaker lung inflammatory response than FA21 and the particle was apparently cleared more rapidly. Our findings are also consistent with previous work demonstrating a strong correlation in pathology and nickel content among a series of nine different MWCNT preparations, where higher nickel particles were found to cause more lung inflammation and pathology than low nickel particles (Hamilton et al., 2012). Notably, these authors demonstrated that MWCNT FA10B, which has low nickel content (2.83% Ni) similar to FA04 and an agglomerate size (405 nm in media) similar to FA21, caused a weak pulmonary inflammatory response (Hamilton et al., 2012). Collectively, these results indicate that the difference in the pro-inflammatory capacity of the MWCNT is more likely due to their nickel content. Moreover, instillation of FA21 but not FA04 was associated with the release of cysLTs, implying the presence of nickel may facilitate the release of such mediators. Nickel associated with MWCNT has been shown to promote inflammasome activation in murine alveolar macrophages (Hamilton et al., 2012). In this respect, FA21, but not FA04, was effective at inducing the release of NLRP3 inflammasome-dependent cytokines (IL-1β and IL-18) by alveolar macrophages (Hamilton et al., 2012). Importantly, inflammasome activation is tightly associated with raised eicosanoid biosynthesis, including the release of leukotrienes (von Moltke et al., 2012). Nevertheless, it is important to note that the nickel levels in the FA04 preparation were reduced rather than the metal being absent (FA04 and FA21 preparations contained 2.54 and 5.54%, respectively). Consequently, any differences in inflammatory effects mediated by nickel is likely to derive from differences in the amount of biologically active or released nickel. Critically, nickel has well characterized pro-inflammatory properties and is a frequent cause of contact sensitization in humans and induction of type IV delayed-type hypersensitivity, in part by activating the toll-like receptor 4 (Schmidt et al., 2010).

It has been previously suggested that MWCNT entering the airways promote the release of high-mobility group box 1 protein (HMGB1) from damaged cells. Interestingly, the extracellular HMGB1 was required for the activation of the NLRP3 inflammasome, and the onset of the eosinophilic inflammatory response. Conceivably, such events likely precede the release of leukotrienes since inflammasome activation is typically a prerequisite for release of these mediators (Jessop and Holian, 2015).

In summary, our study demonstrated that inhalation of high nickel MWCNT promotes a rapid lung eosinophilic inflammation and causes an intense exacerbation of pre-existing allergic airway inflammation. The augmented inflammatory response was not only associated with, but also dependent on, the production of leukotrienes in response to the nanomaterials. These findings suggest that exposure to airborne MWCNT is likely to have adverse health effects in individuals suffering from atopic asthma and merit further investigation of the potential therapeutic effects of pharmacological agents that block leukotriene biosynthesis.

## Ethics Statement

All animal experiments in this study were approved by the Institutional Animal Care and Use Committee (IACUC), University of Montana, and performed in accordance with the approved guidelines set forth by IACUC.

## Author Contributions

SC, MF, LH, AH, ZJ, and KR conceived, designed, or performed the experiments. KR provided overall coordination and supervision of the study. SC, ZJ, and KR analyzed the data and wrote the manuscript.

## Conflict of Interest Statement

The authors declare that the research was conducted in the absence of any commercial or financial relationships that could be construed as a potential conflict of interest.
