# Supplementary material for: Multi-Walled Carbon Nanotubes Augment Allergic Airway Eosinophilic Inflammation by Promoting Cysteinyl Leukotriene Production
Source: Front Pharmacol. 2018 Jun 5;9:585. doi: 10.3389/fphar.2018.00585 (PMC5996183; doi:10.3389/fphar.2018.00585)
Supplement: Supplementary file 1 [file Image_1.PDF]

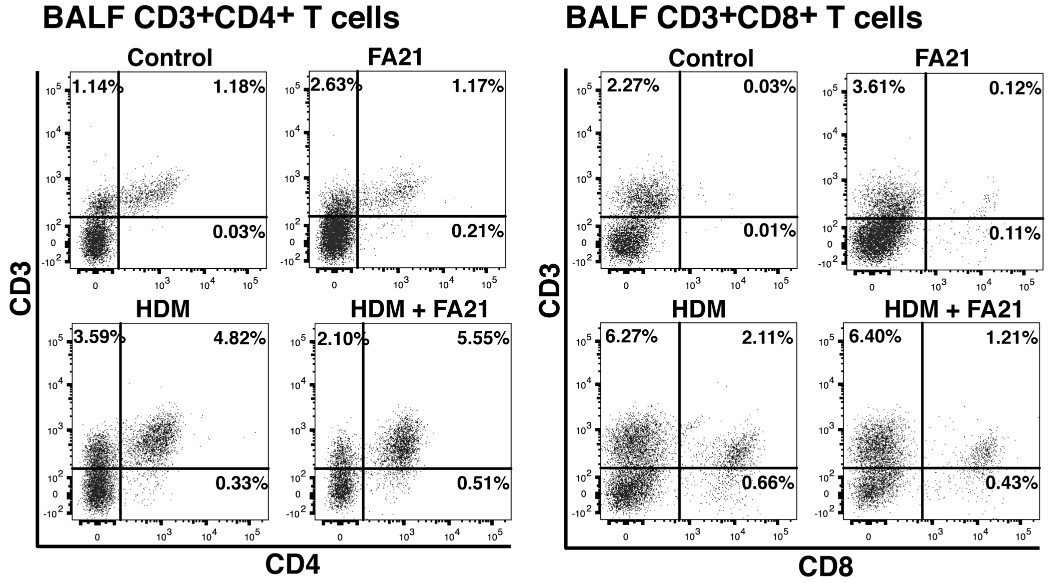

Supplemental Figure 1. Intratracheal administration of MWCNT did not result in any changes in the number CD4$^+$ or CD8$^+$ T cells present in the airways of allergen challenged mice. C57BL/6 mice (4 per group) were intranasally challenged with PBS (control) or HDM allergen over a two-week period (on days 0, 7 and 14) to induce allergic inflammation and then exposed to vehicle or FA21 MWCNT (50 μg) on day 15 by intratracheal administration. Groups comprised of mice treated with FA21 alone, HDM alone, HDM + FA21 or carrier alone (control). BALF was collected 24 hours after MWCNT administration (on day 16) and the effect on the number of airway CD3$^+$CD4$^+$ and CD8$^+$ T cells was analyzed by flow cytometry. Data are representative of 3 independent experiments.
